# Expression of HIV-1 Vpu Leads to Loss of the Viral Restriction Factor CD317/Tetherin from Lipid Rafts and Its Enhanced Lysosomal Degradation

**DOI:** 10.1371/journal.pone.0075680

**Published:** 2013-09-24

**Authors:** Ruth Rollason, Katie Dunstan, Peter G. Billcliff, Paul Bishop, Paul Gleeson, Helen Wise, Paul Digard, George Banting

**Affiliations:** 1 School of Biochemistry, University of Bristol, Bristol, United Kingdom; 2 Bio21 Institute, University of Melbourne, Melbourne, Australia; 3 The Roslin Institute, University of Edinburgh, Edinburgh, United Kingdom; Institute of Molecular and Cell Biology, Biopolis, United States of America

## Abstract

CD317/tetherin (aka BST2 or HM1.24 antigen) is an interferon inducible membrane protein present in regions of the lipid bilayer enriched in sphingolipids and cholesterol (often termed lipid rafts). It has been implicated in an eclectic mix of cellular processes including, most notably, the retention of fully formed viral particles at the surface of cells infected with HIV and other enveloped viruses. Expression of the HIV viral accessory protein Vpu has been shown to lead to intracellular sequestration and degradation of tetherin, thereby counteracting the inhibition of viral release. There is evidence that tetherin interacts directly with Vpu, but it remains unclear where in the cell this interaction occurs or if Vpu expression affects the lipid raft localisation of tetherin. We have addressed these points using biochemical and cell imaging approaches focused on endogenous rather than ectopically over-expressed tetherin. We find i) no evidence for an interaction between Vpu and endogenous tetherin at the cell surface, ii) the vast majority of endogenous tetherin that is at the cell surface in control cells is in lipid rafts, iii) internalised tetherin is present in non-raft fractions, iv) expression of Vpu in cells expressing endogenous tetherin leads to the loss of tetherin from lipid rafts, v) internalised tetherin enters early endosomes, and late endosomes, in both control cells and cells expressing Vpu, but the proportion of tetherin molecules destined for degradation rather than recycling is increased in cells expressing Vpu vi) lysosomes are the primary site for degradation of endogenous tetherin in cells expressing Vpu. Our studies underlie the importance of studying endogenous tetherin and let us propose a model in which Vpu intercepts newly internalised tetherin and diverts it for lysosomal destruction rather than recycling to the cell surface.

## Introduction

CD317/tetherin (aka BST2 or HM1.24 antigen [1,2]) is an interferon inducible membrane protein [3] that causes retention of fully formed viral particles at the surface of HIV infected cells [4,5]. In fact it has been shown to restrict the release of a range of enveloped viruses from infected cells (reviewed in [6]) as well as having been implicated in an eclectic mix of cellular processes (summarized in [7]).

Tetherin possesses both a conventional transmembrane (TM) domain and a glycosylphosphatidylinositol (GPI) anchor [8]. The presence of a GPI anchor has been shown by both biochemical means [8] and by a targeted proteomics approach [9] and is consistent with studies in a CHO cell line deficient in the enzyme required for the addition of GPI anchors [10]; however a recent report suggests that the C-terminal hydrophobic region of tetherin serves as a second TM domain rather than as a signal for the addition of a GPI anchor [11]. Tetherin resides – at least at the cell surface – in lipid rafts (membrane microdomains in which there is a ‘preferential association between sphingolipids, sterols, and specific proteins’ [12,13]) with the TM domain apparently lying outside the raft (or at the interface of the raft and non-raft domains) and with the raft localisation being dependent upon the GPI anchor [8,14]. The extracellular domain of tetherin has been shown to form a disulphide bonded parallel coiled coil, thereby generating a dimer with two adjacent TM domains and two adjacent GPI anchors separated by ~17nm [15,16,17,18]. It has been suggested that the structure of tetherin plays a role in the mechanism by which it restricts the release of newly formed viral particles from infected cells [15,16,17,18].

Several enveloped viruses have evolved specific mechanisms to counteract the restriction imposed by tetherin. This generally involves expression of a viral protein which interacts with tetherin (e.g. Ebola virus GP) [19,20], in some cases leading to the degradation of tetherin (e.g. the K5 ubiquitin ligase of Kaposi’s sarcoma-associated herpesvirus) [21]. In the case of HIV-1, it is the viral accessory protein Vpu that has been shown to antagonise tetherin [5,22,23,24]. Vpu is a member of a family of viral proteins, termed viroporins, that oligomerise to form channels in membranes [25]. Vpu has a single TM domain, but oligomerises to form a pentameric ion channel in the membrane [26,27]. The precise mechanism by which Vpu antagonizes tetherin remains unclear, as there are conflicting data in the literature (reviewed in [6,24,28]).

Tetherin and Vpu have been shown to interact, with this interaction being dependent upon residues within the TM domains of the two proteins, principally residues located at the extracellular ends of their TM domains [23,29,30,31,32,33,34]. However the stoichiometry and organisation of this interaction has not been characterized, i.e. does each monomer in a Vpu tetramer/pentamer interact with a tetherin dimer or is there some other arrangement? What is known is that mutations which abrogate the interaction between tetherin and Vpu restore the capacity of tetherin to restrict viral release (reviewed in [28]). The interaction between tetherin and Vpu leads to the degradation of tetherin, with some dispute as to whether this is primarily via a lysosomal or proteasomal mediated pathway (reviewed in [6,24,28]) but with the more recent and more compelling evidence being in favour of lysosomal degradation [35,36,37]. However the degradation of tetherin does not appear to be an absolute requirement for overcoming its restriction of viral release [38,39], with several reports of Vpu-mediated sequestration of tetherin in the trans Golgi network (TGN) being sufficient [36,37,40,41] and at least one report that the primary antagonistic effect of Vpu on tetherin is upon newly synthesised tetherin as the latter is being transported to the cell surface [42]. It is of note though, given the lipid raft localisation of tetherin, that lipid raft association of Vpu has been shown to correlate with enhanced virus release [43]. Indeed, Dube et al comment ‘It will be important to determine whether Vpu targets a pool of Tetherin located in specific microdomains of the plasma membrane’ [6].

There have been reports of the interaction between tetherin and Vpu occurring at various cellular locations, including the endoplasmic reticulum (ER) [42], TGN [40] and the cell surface [44,45]. However, the latter studies provide no direct evidence for the tetherin:Vpu interaction occurring at the cell surface. Interpretation of data from studies on the effects of Vpu on tetherin have been complicated by the fact that many have monitored epitope-tagged tetherin expressed in cells that do not express endogenous tetherin (HEK293T cells are most commonly used). Notably, the different forms of tetherin (immature, fully glycosylated etc.) are present in different proportions in transiently transfected cells compared to the endogenous protein (e.g. [24]). Even minor changes to the tetherin sequence have been shown to compromise the efficiency with which it restricts virion release (reviewed in [46]), making it difficult to predict the effect of an epitope tag (such as is commonly used when recombinant tetherin is expressed in cells) upon the Vpu-mediated fate of tetherin. We therefore set out to investigate the effects of Vpu expression on the localisation and fate of endogenous tetherin in HeLa cells.

## Results

### Do Vpu and tetherin interact at the cell surface?

**Figure 1:**
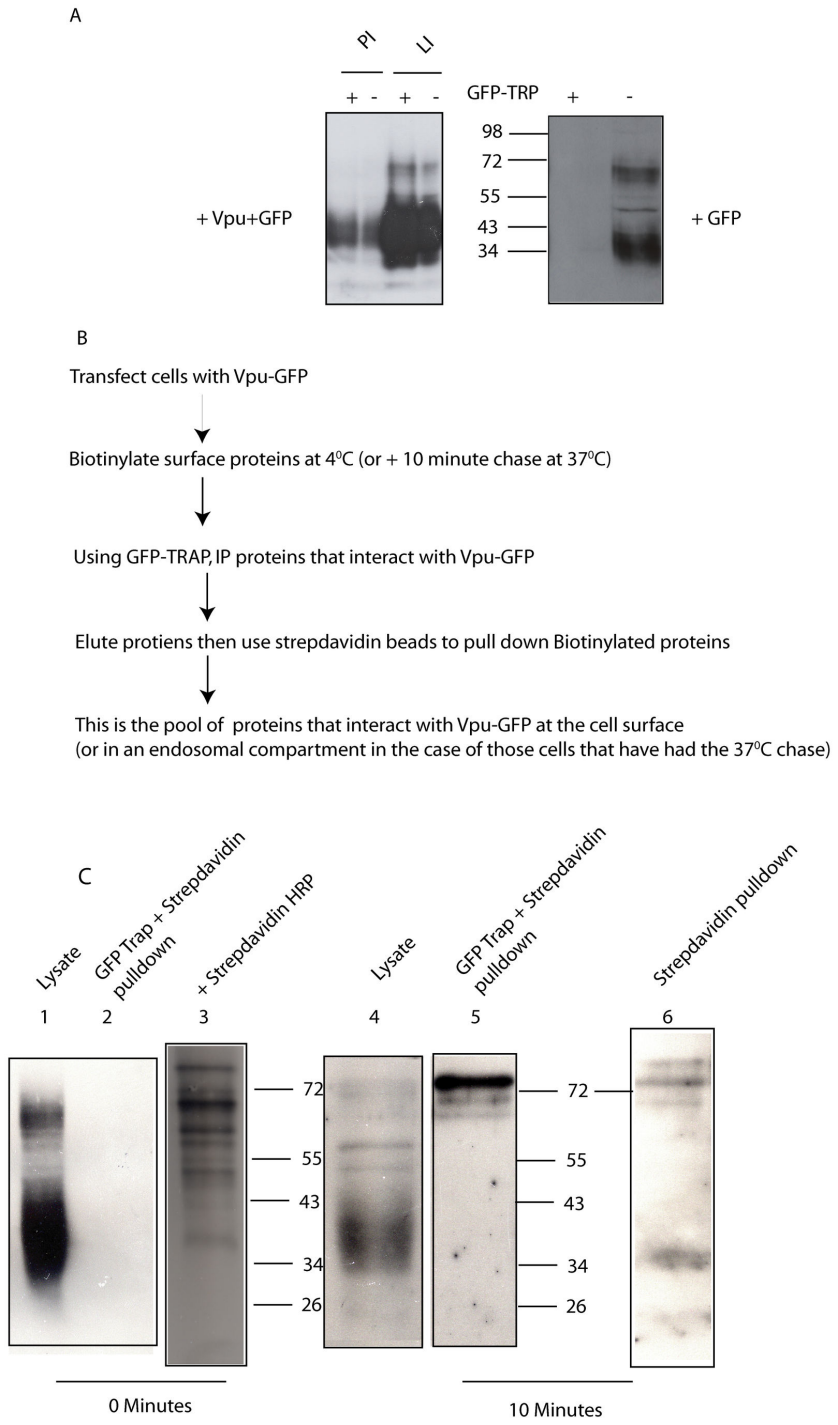
Tetherin interacts with Vpu in whole cells, but does not do so at the cell surface. **A**. Immunoblot of tetherin in whole cell lysates from HeLa cells transfected to express Vpu-GFP (left panel) or GFP alone (right panel) and incubated in the presence of proteosomal inhibitors (PI) or lysosomal inhibitors (LI) as indicated. These inhibitors were included in order to block any Vpu-mediated degradation of tetherin, thereby ensuring sufficient tetherin remains for detection by immunoblot. Tetherin was detected in whole cell lysates (10% of input; GFP-TRP – lanes) and in co-immunoprecipitates with Vpu-GFP, but not in co-immunoprecipitates with GFP (GFP-TRP + lanes). **B**. Flow diagram to illustrate procedure for isolation and identification of any biotinylated tetherin that interacts with Vpu, either at the cell surface (biotinylation at 4°C) or in an endosomal compartment following internalization from the cell surface (biotinylation at 4°C followed by a 10 min chase at 37°C). **C**. HeLa cells were processed as outlined in B (above) and samples were processed for immunoblot analysis using an antibody to tetherin. Tetherin is detected in the whole cell lysate (10% of input) from cells labeled with biotin at 4°C (0 min) (lane 1) and from cells labeled with biotin at 4°C followed by a 10 min chase at 37°C (10 min) (lane 4). Tetherin is not detected in the GFP Trap and streptavidin pull-down from cells labeled with biotin at 4°C (0 min) (lane 2), indicating that cell surface tetherin does not interact with Vpu. Multiple cell surface proteins were biotinylated by the procedure used, as shown by their detection in the whole cell lysate using streptavidin HRP (lane 3). Tetherin is detected in the GFP Trap and streptavidin pull-down from cells labeled with biotin at 4°C then subjected to a 10 min chase at 37°C (10 min) (lane 5), indicating that tetherin does interact with Vpu upon internalisation. Tetherin can be detected in material isolated from the 10 min chase lysate by streptavidin-coated beads (lane 6).

We initially chose to confirm that we could co-immunoprecipitate endogenous tetherin with Vpu. HeLa cells (which express endogenous tetherin) were transiently transfected to express GFP-tagged Vpu and, 24 hours post transfection, cells were lysed and processed for immunoprecipitation using the GFP-trap monoclonal antibody (see Methods). As anticipated, tetherin was efficiently co-immunoprecipitated with Vpu (Figure 1A).

It has been suggested, but not directly demonstrated, that the Vpu-tetherin interaction can occur at the cell surface [44,45]. We tested this directly by using a cell surface biotinylation and co-immunoprecipitation approach. HeLa cells were transiently transfected to express GFP-tagged Vpu and, 24hrs later, labeled with a membrane impermeant biotin reagent at 4°C (see Methods). This procedure labels only the extracellular domains of proteins (on lysine residues) exposed at the cell surface and the low temperature will prevent their internalisation. Excess biotin was then washed away (at 4°C) and aliquots of cells either immediately processed for immunoprecipitation, or transferred to 37°C for various periods of time before being processed for immunoprecipitation, using the GFP-trap monoclonal antibody. Endocytosis of cell surface proteins occurs following the increase in temperature to 37°C, and therefore allows one to follow the intermolecular interactions of biotinylated proteins that have been at the cell surface following their internalisation. Immunoprecipitated material was then separated into that which bound to strepatavidin-agarose (corresponding to the biotinylated proteins) and that which did not (i.e. non-biotinylated proteins). The different fractions were then subjected to SDS-PAGE and immunoblot analysis using an anti-tetherin antibody as primary antibody (see flow diagram in Figure 1B). No biotinylated tetherin could be detected in the GFP-trap immunoprecipitate of lysate from cells that had been kept at 4°C (Figure 1C lane 2), indicating a lack of detectable interaction between tetherin and Vpu at the cell surface. However, biotinylated tetherin could be detected in the GFP-trap immunoprecipitate of lysate from cells that had been incubated at 37°C for 10 min following labeling at 4°C (Figure 1C lane 5). This suggests that either a) tetherin and Vpu do interact at the cell surface, but that their internalisation occurs as soon as they have interacted, or b) the interaction between Vpu and tetherin occurs very early in the endocytic pathway. It is of note that it is a high molecular weight (~75kDa) form of biotinylated tetherin that co-immunoprecipitates with Vpu-GFP following internalisation of the biotinylated tetherin from the cell surface. This potentially represents an SDS-resistant dimer of tetherin and implies preferential interaction of this form of tetherin with Vpu-GFP. Not all of the biotinylated tetherin in lysate from cells that had been incubated at 37°C for 10 min following labeling at 4°C is present as an SDS-resistant dimer, as shown by the fact that a lower molecular weight form of tetherin can be isolated from this lysate using streptavidin beads (Figure 1C lane 6)

### Does expression of Vpu affect the distribution of tetherin at the cell surface?

**Figure 2:**
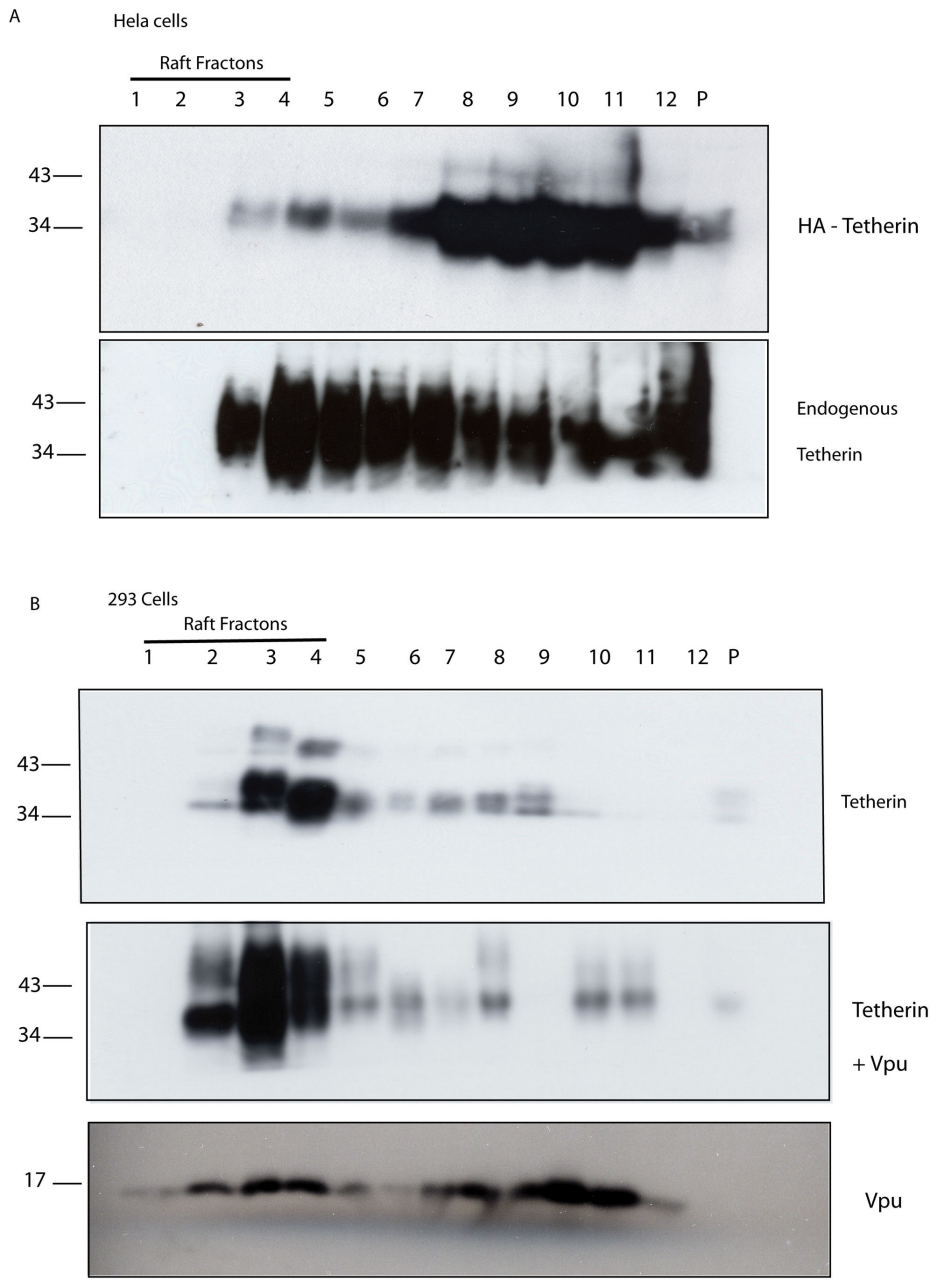
Variation in the degree of localization of tetherin to lipid rafts. Cells were subjected to extraction in ice-cold Triton X-100 (1%) prior to separation by sucrose density gradient centrifugation, then analysis of fractions from the gradients by immunoblot. Fractions 1-4 are considered as the lipid raft fraction, fractions 5-12 as non-raft fractions. A. The top panel shows an immunoblot, using an antibody to the HA epitope tag on recombinant HA-tagged tetherin expressed in HeLa cells, of fractions from HeLa cells transfected to express HA-tagged tetherin and shows that the majority of recombinant HA-tagged tetherin is not present in lipid rafts (i.e. it is in fractions 5-12). The second panel shows an immunoblot, using an antibody to tetherin, of fractions from HeLa cells and shows that a significant proportion of endogenous tetherin is present in lipid rafts (i.e. in fractions 1-4). B. The first panel shows an immunoblot, using an antibody to the HA epitope tag on recombinant HA-tagged tetherin expressed in HEK293T cells (NB HEK293T cells do not express detectable levels of endogenous tetherin), of fractions from HEK293T cells transfected to express HA-tagged tetherin and shows that the majority of recombinant tetherin is present in lipid rafts (i.e. in fractions 1-4). The second panel shows the same, but in HEK293T cells that express Vpu-GFP in addition to HA-tagged tetherin: this experiment was performed in the absence of lysosomal enzyme inhibitors, the blot was exposed longer to ensure detection of tetherin. The bottom panel shows the distribution of Vpu-GFP expressed in the same cells; it is present in both raft and non-raft fractions.

We next asked whether expression of Vpu affects the distribution of endogenous tetherin in the plasma membrane. First, we tested the association of tetherin with lipid rafts through membrane flotation assays. Untransfected HeLa cells, or cells transfected to express an HA-tagged tetherin construct, were lysed in ice-cold 1% Triton X-100 and separated on sucrose density gradients before immunoblotting with antibodies to either tetherin (for the endogenous protein) or the HA tag (to detect the transfected protein) respectively. Some, but not all of the endogenous tetherin is present in the low density fractions representing lipid rafts (Figure 2, second panel), as we have shown previously [7,8]. This is consistent with the current view of the plasticity of membrane microdomains at the cell surface [12,13]. However, when recombinant tetherin is expressed in HeLa cells a significant proportion is not present in lipid rafts, implying that the rafts have become saturated for tetherin [7] and (Figure 2 upper panel). In contrast, when recombinant tetherin is expressed in HEK293T cells (chosen because they do not express detectable levels of endogenous tetherin and are frequently used as a background in which to express recombinant tetherin constructs), the majority of the tetherin molecules are present in lipid rafts (Figure 2B), a finding that was not altered by the co-expression of Vpu (Figure 2B lower panels). These observations of differential behaviour between endogenous and recombinant tetherin re-enforced our rationale for investigating the relationship between endogenous tetherin and Vpu.

**Figure 3:**
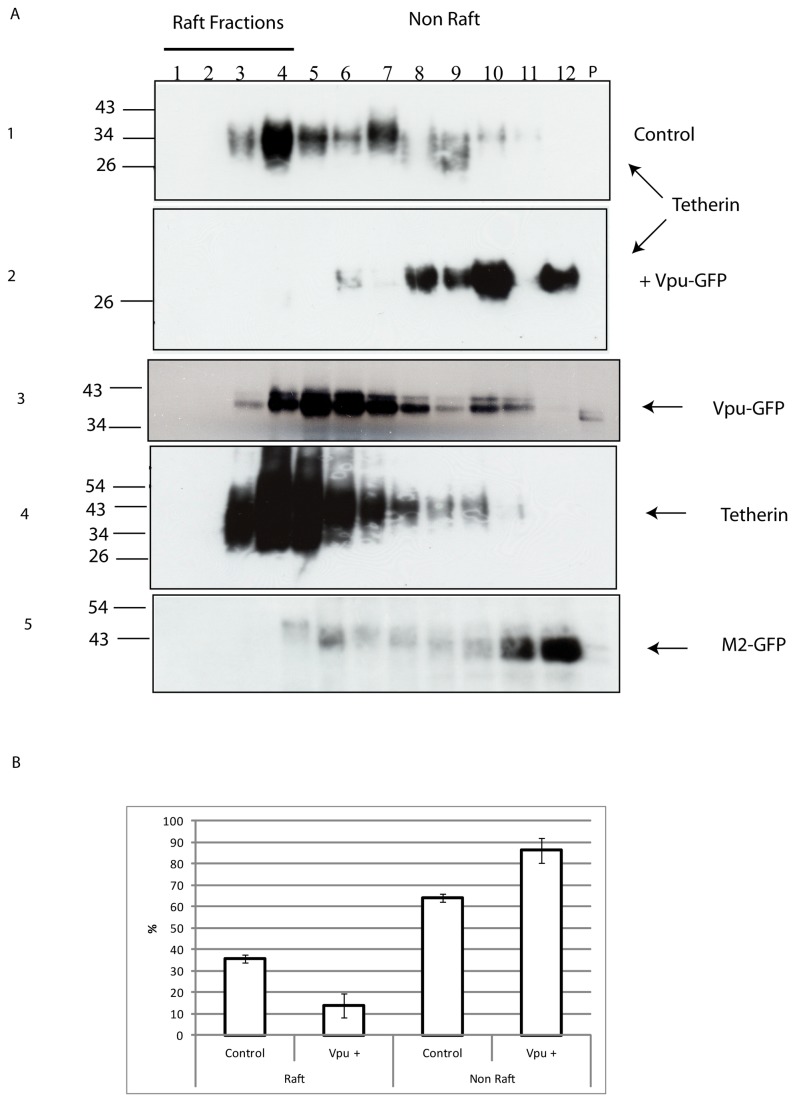
The effect of expression of Vpu on the lipid raft localisation of endogenous tetherin. **A**. HeLa cells were subjected to extraction in ice-cold Triton X-100 (1%) prior to separation by sucrose density gradient centrifugation, then analysis of fractions from the gradients by immunoblot. Fractions 1-4 are considered as lipid raft fractions, fractions 5-12 as non-raft fractions. The top panel shows an immunoblot, using an antibody to tetherin, of fractions from untransfected HeLa cells and confirms that a significant proportion of endogenous tetherin is present in lipid rafts (i.e. in fractions 1-4). The second panel shows an immunoblot, using an antibody to tetherin, of fractions from HeLa cells that had been transfected to express Vpu-GFP and shows that the majority of endogenous tetherin is lost from lipid rafts (i.e. fractions 1-4) in the presence of Vpu-GFP: this experiment was performed in the absence of lysosomal enzyme inhibitors, the blot was exposed longer to ensure detection of tetherin. Panel 3 shows the distribution of Vpu-GFP across the sucrose gradient; it has a broad distribution across the gradient, indicating that it is present in a range of membrane environments, both raft and non-raft. Expression of another viroporin protein (the M2 protein of influenza virus) does not lead to a redistribution of tetherin from lipid rafts, as shown by the bottom two panels. Panel 4 shows an immunoblot, using an antibody to tetherin, of fractions from HeLa cells that had been transfected to express M2-GFP and shows that the distribution of endogenous tetherin is similar to that in non-transfected cells (top panel). The bottom panel shows the distribution of M2-GFP across the sucrose gradient. **B**. Graphical representation of the proportions of tetherin molecules in raft vs. non-raft fractions on control HeLa cell and HeLa cells expressing Vpu-GFP (as indicated, n=5).

To test the effect of Vpu on the raft-association of endogenous tetherin, control HeLa cells and HeLa cells that had been transfected to transiently express Vpu-GFP were lysed in ice cold Triton X-100 and separated on sucrose density gradients. Fractions from the gradients were then subjected to SDS-PAGE and immunoblot analysis. A proportion of mature tetherin molecules is present in the raft fractions (1-4) in lysate from control cells, but a substantially lower proportion is raft localised in lysate from cells expressing Vpu (Figure 3A panels 1+2). When the proportion of endogenous tetherin present in raft vs. non-raft fractions was quantified (by densitometry) 36 +/- 4% of tetherin molecules were detected in raft fractions in control cells, while expression of Vpu-GFP lowered this to only 14 +/- 3% (Figure 3B, n = 5). Similar results were obtained using cells expressing untagged Vpu (data not shown). In contrast, when the effect of Vpu was tested on recombinant tetherin expressed in HEK293T cells, co-expression of Vpu did not lead to redistribution of recombinant tetherin to non-raft fractions (Figure 2B). The distribution of Vpu-GFP across the sucrose gradient was also analysed by immunoblotting; in both cell types it has a broad distribution across the gradient (Figure 2B, bottom panel, Figure 3A panel 3), indicating that it is present in a range of membrane environments, both raft and non-raft. The effect of Vpu-GFP expression on the raft vs. non-raft distribution of endogenous tetherin is not a generic effect of viroporin expression, as expression of the Influenza virus M2 protein (another viroporin) had no effect on the raft localisation of tetherin (Figure 3A panels 4+5).

Thus, expression of Vpu correlates with a redistribution of endogenous tetherin from raft to non-raft membrane environments, but this does not necessarily mean that a direct interaction between the two proteins is the mechanism by which tetherin is removed from lipid rafts.

### What is the sub-cellular location of tetherin that is in lipid rafts?

**Figure 4:**
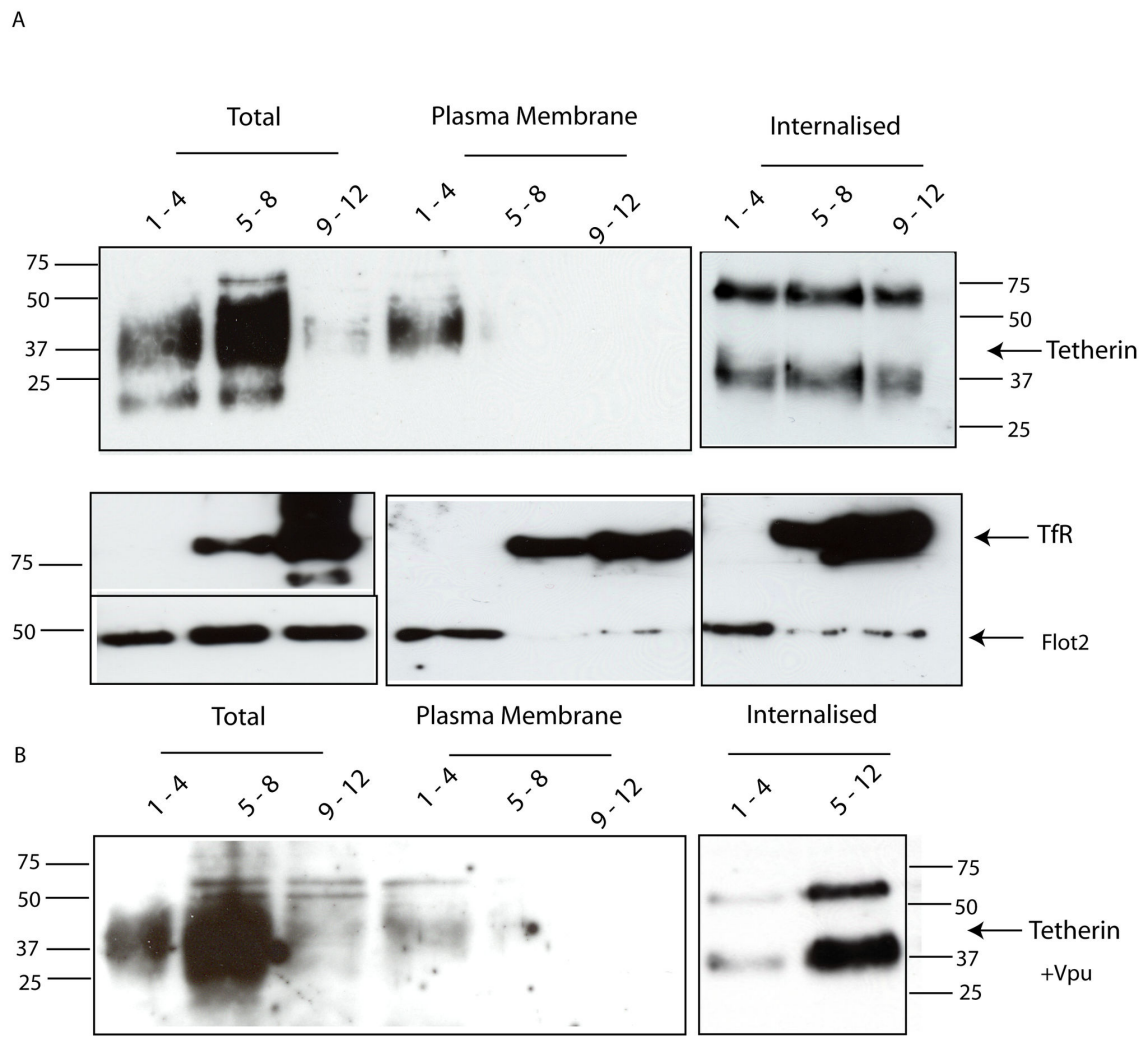
Tetherin is in lipid rafts at the cell surface. **A**. HeLa cells were labeled with a membrane impermeant biotin reagent at 4°C prior to either immediate lysis in ice cold Triton X-100, or incubation for 10 min at 37°C before lysis in ice cold Triton X-100. Lysates were then separated on sucrose density gradients and 1 ml fractions were taken. Fractions 1-4 were pooled as raft fractions, 5-8 and 9-12 as two separate non-raft fractions. Lysates were then incubated with streptavidin-agarose beads to separate biotinylated (cell surface in the case of immediate lysis after biotinylation at 4°C, or internalised in the case of the 10 min chase at 37°C, proteins) from non-biotinylated proteins. Total, plasma membrane, and internalised fractions were then subjected to immunoblot analysis using antibodies to endogenous tetherin, Transferrin receptor or Flotillin 2 (the latter two as controls) as indicated. The total population of tetherin molecules is ~ 35% raft localized (1-4 in Total), however the tetherin that is at the cell surface (i.e. the biotinylated tetherin at 0 min uptake at 37°C) is almost exclusively raft localized (1-4 in Plasma Membrane). After 10 min uptake at 37°C a significant proportion of biotinylated tetherin is present in non-raft fractions (Figure 4A Internalised). **B**. As in A, but using HeLa cells transfected to express Vpu-GFP.

The preceding result led us to question whether the tetherin that is in lipid rafts in control cells is at the cell surface or in intracellular compartments (N.B. tetherin constitutively cycles between the cell surface and an intracellular pool [8,47]). HeLa cells were again labeled with a membrane impermeant biotin reagent at 4°C prior to either immediate lysis in ice cold 1% Triton X-100, or incubation for 10 min at 37°C before lysis in ice cold 1% Triton X-100. Lysates were then separated on sucrose density gradients as before. Fractions 1-4 were pooled as raft fractions, and 5-8 and 9-12 as two separate non-raft fractions. Lysates were then incubated with streptavidin-agarose beads to separate biotinylated (either cell surface in the case of immediate lysis after biotinylation at 4°C, or internalised after a 10 min chase at 37°C, proteins) from non-biotinylated material. As expected the total population of tetherin molecules is ~ 35% raft localized (Figure 4A ‘Total’), however the tetherin that is at the cell surface (i.e. the biotinylated tetherin at 0 min uptake at 37°C) is almost exclusively raft localized (Figure 4A ‘Plasma Membrane’). After 10 min uptake at 37°C a significant proportion of biotinylated tetherin is present in non-raft fractions (Figure 4A ‘Internalised’). Since it has been shown that not all tetherin molecules will have been internalised in 10 min [36,40], it is probable that the fraction of tetherin that remains raft associated following 10 min incubation at 37°C is still at the cell surface. Thus, these data are consistent with tetherin exiting lipid rafts upon, or immediately prior to, internalisation. Expression of Vpu has no significant effect on this distribution pattern (Figure 4B), implying that the preferential redistribution of the total population of tetherin molecules to non-raft fractions upon expression of Vpu in cells (as observed in Figure 3) is in fact a consequence of a reduction in the levels of tetherin at the cell surface in the presence of Vpu (as has been described previously, e.g. [5]) rather than a Vpu-mediated lateral movement of tetherin out of lipid rafts in the plane of the lipid bilayer of the plasma membrane. In contrast to the situation with tetherin, most Transferrin receptor molecules are in the non-raft fractions and most Flotillin-2 molecules are in the raft fractions, under all conditions assayed (Figure 4A lower panels).

It is of note that the internalised tetherin is detected as bands corresponding to both monomeric and dimeric forms of tetherin (Figure 4A and B ‘Internalised’). This is under reducing conditions and in the presence of SDS, and is consistent with the presence of a tetherin dimer that is resistant to both reducing conditions and to SDS (as observed to be preferentially associating with Vpu in Figure 1C). The bands corresponding to tetherin detected in Figure 4B (i.e. the internalised tetherin that co-immunoprecipitates with Vpu) are also less diffuse than the corresponding bands in Figure 4A (i.e. the total population of internalised tetherin molecules); this might indicate that Vpu preferentially interacts with a particular glycoform (or subset of glycoforms) of the heterogeneously glycosylated tetherin.

### What is the fate of endogenous tetherin in cells expressing Vpu?

**Figure 5:**
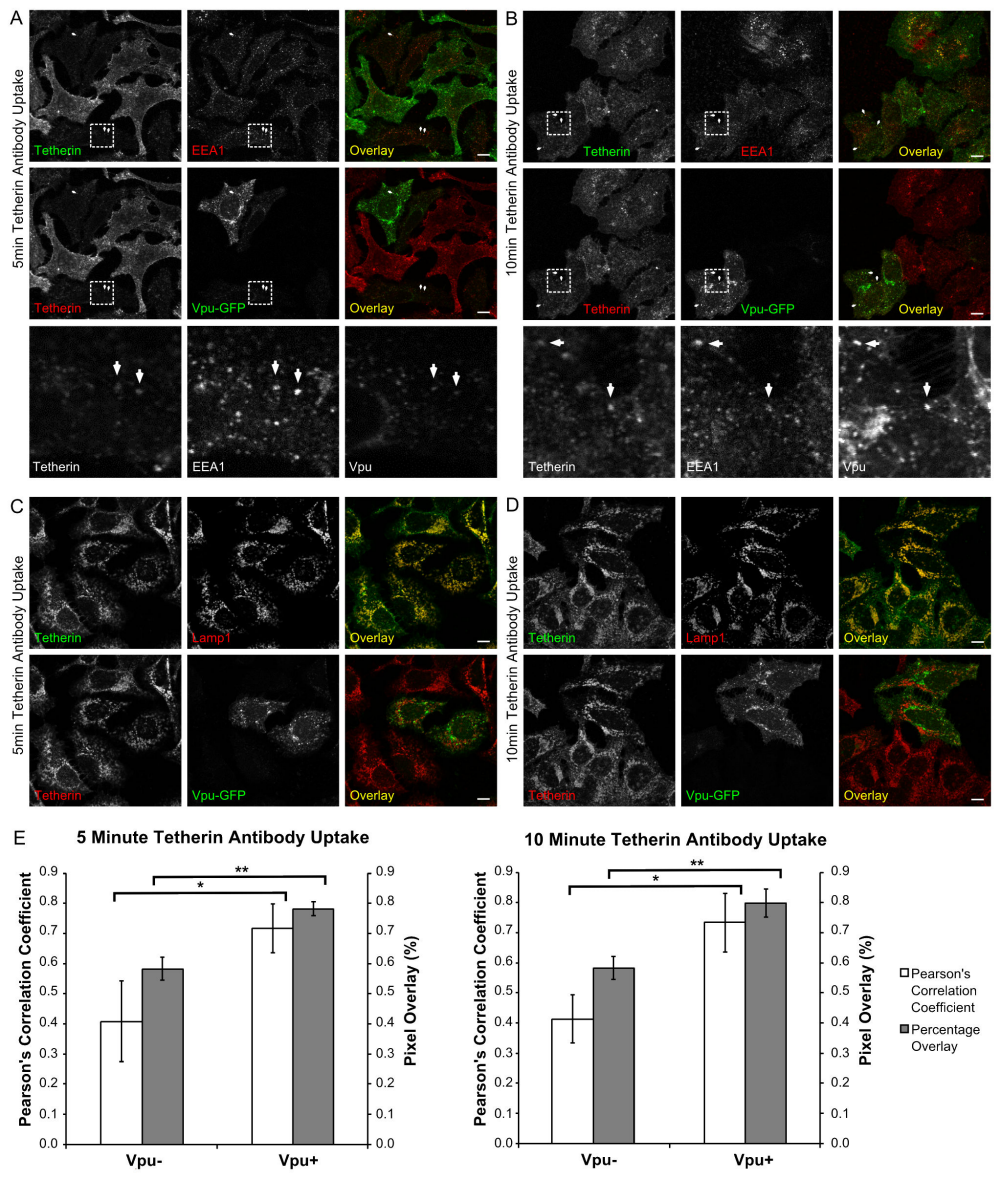
Immunolocalisation of internalized tetherin in the presence and absence of Vpu. **A**-**D**. HeLa cells that had been transiently transfected to express Vpu-GFP and which had been incubated in the presence of lysosomal enzyme inhibitors for 24 hours were incubated at 4°C for 60 min with a monoclonal antibody (HM1.24) that recognises the extracellular domain of tetherin. Cells were then transferred to 37°C for various times and processed for immunofluorescence analysis using polyclonal antibodies to markers of early (EEA-1, **A, B**) or late (LAMP-1, **C, D**) endosomes. Some of the internalised tetherin can be seen to colocalise with both Vpu and EEA1 after 5 (**A**) or 10 (**B**) min at 37°C (see white arrows; bottom sets of panels represent magnifications of the boxed regions from the corresponding images above and are 20µ^2^); however, there is greater evidence of colocalisation between internalized tetherin and LAMP-1 at both time points (**C** and **D**). This is quantified by Pearson’s correlation coefficient (PCC) and percentage pixel overlay (%O) in **E**. This co-localisation is greatest in cells that also express Vpu at both 5min (PCC P=0.027, %O P=0.0015 n=3) and 10min (PCC P=0.0117, %O P=0.0034 n=3) antibody uptake. It is of note that there is limited evidence of any co-localisation between Vpu and LAMP-1 (**C** and **D**). Bar = 10µ.

Given that the co-immunoprecipitation data presented in Figure 1 are consistent with Vpu interacting with tetherin immediately following the internalisation of tetherin from the cell surface, we tested if Vpu and endocytosed tetherin could be detected in a common endocytic compartment. In order to do this, HeLa cells that had been transiently transfected to express Vpu-GFP and which had been incubated in the presence of lysosomal enzyme inhibitors (see Methods) for 24 hours were incubated for 30 min at 4°C in order to halt endocytosis, and then incubated for a further 15 min at 4°C with a monoclonal antibody (HM1.24) that recognises the extracellular domain of tetherin. Cells were then transferred to 37°C for various times and processed for immunofluorescence analysis (see Methods) using polyclonal antibodies to markers of early (EEA-1) or late (LAMP-1) endosomes. Some of the internalised tetherin can be seen to co-localise with both Vpu and EEA1 after 5 or 10 min at 37°C (Figure 5A and B, white arrows). However, there is greater evidence of co-localisation between internalised tetherin and LAMP-1 at both time points (Figure 5C and D). This co-localisation is greatest in cells that also express Vpu (quantified in Figure 5E), but it is of note that there is limited evidence of any co-localisation between Vpu and LAMP-1 (Figure 5C and D) implying that the presence of Vpu leads to increased delivery of internalised tetherin to late endosomes without Vpu itself trafficking to that compartment. NB the images shown in Figure 5 represent the steady-state distribution of EEA-1, LAMP-1 and Vpu-GFP, but only the internalised population of tetherin molecules.

**Figure 6:**
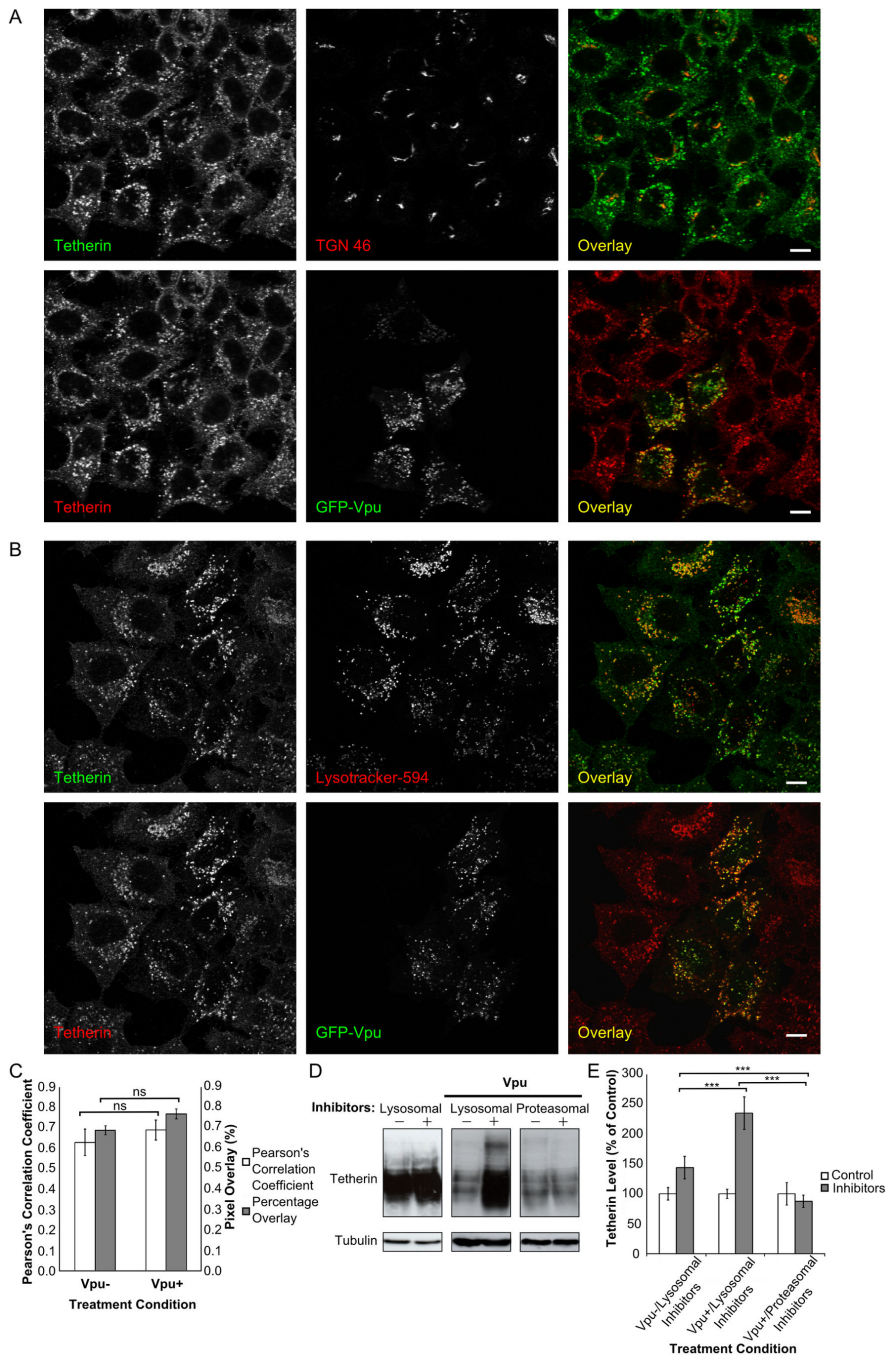
Lysosomal degradation of tetherin. **A**.and **B**. Triple label fluorescence analysis (antibody detection of Vpu, tetherin and TGN46, and LysoTracker detection of lysosomes) of HeLa cells that had been incubated in the presence of lysosomal inhibitors for 24 hours prior to processing for immunofluorescence analysis. Cells expressing Vpu demonstrated significant accumulation of tetherin in lysosomes (**B**), but not in the TGN (**A**). Bar = 10µ. **C**. Quantification of co-localisation between LysoTracker-594 and tetherin, using both Pearson’s correlation coefficient and percentage pixel overlay. **D**. Immunoblot analysis, using an antibody to endogenous tetherin, of lysates from control HeLa cells, or HeLa cells expressing Vpu-GFP (Vpu) that had been incubated in the presence of lysosomal (24 hours) or proteosomal (12 hours) inhibitors (as indicated). The lower panels show immunoblots for tubulin as loading controls. **E**. Graphical representation of quantification of the data presented in **D**, P=0.0002, n = 3.

To extend our investigation of the fate of internalised tetherin in the presence of Vpu, we undertook triple label fluorescence analysis (detecting Vpu, tetherin and the TGN or lysosomes) of HeLa cells that had been incubated in the presence of lysosomal inhibitors for 24 hours prior to processing for fluorescent labelling. This demonstrated minimal accumulation of tetherin in the TGN (Figure 6A), but significant accumulation in lysosomes in both control cells and in expressing Vpu (Figure 6B, quantified in Figure 6C). The fact that the majority of endogenous tetherin is targeted for lysosomal degradation in cells expressing Vpu was confirmed by immunoblot analysis of lysates from cells that had been incubated in the presence of lysosomal inhibitors (Figure 6D). In the absence of Vpu, inhibition of lysosomal enzymes led to a small increase in tetherin levels (left hand panel in Figure 6D; quantification in Figure 6E), indicating tetherin is normally susceptible to lysosomal degradation (this is consistent with the reported short half-life of tetherin [40]). However, in cells expressing Vpu, inhibition of lysosomal enzymes caused a much more substantial (> two fold; Figure 6D and E) increase in tetherin accumulation (compare central panel with left hand panel of Figure 6D). In contrast, proteosomal inhibitors have a minimal effect on endogenous tetherin levels whether or not cells express Vpu (Figure 6D right hand panel, Figure 6E and data not shown). Overall this confirms that Vpu directs the lysosomal degradation of internalised tetherin.

## Discussion

**Figure 7:**
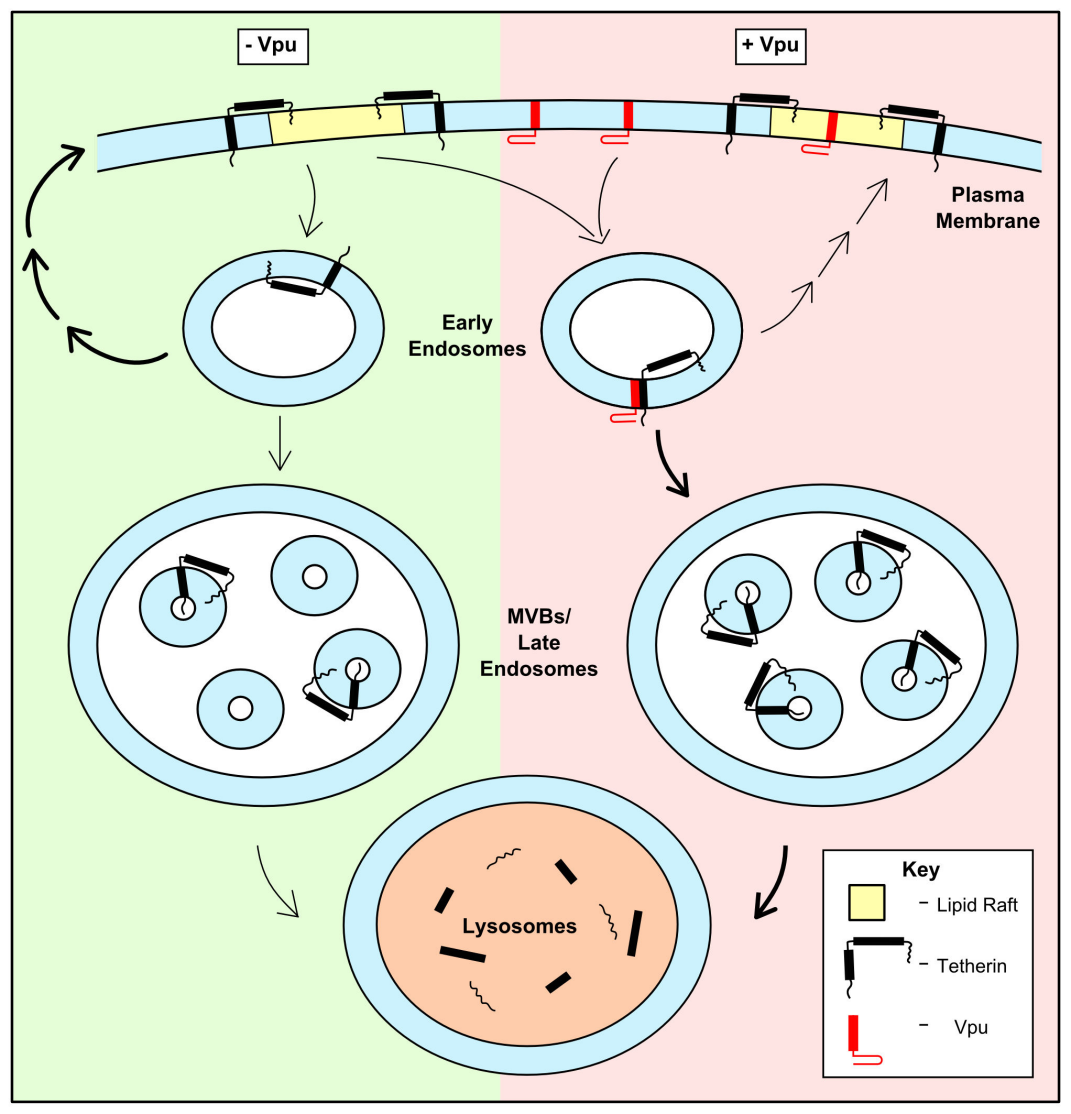
Cartoon representation of the effect of Vpu on tetherin trafficking following internalisation from the cell surface. The left hand side of the cartoon (green) shows what happens to cell surface tetherin in the absence of Vpu. It is internalised and delivered to early endosomes. During this process it exits lipid rafts. The majority of tetherin molecules then end up being recycled to the cell surface, probably via one or more intermediate compartment (as indicated by the thick arrows) whilst some tetherin ends up in multivesicular bodies (MVBs)/late endosomes and is ultimately destined for lysosomal degradation. Thus the fate of internalised tetherin is finely balanced between recycling to the cell surface and lysosomal degradation. The right hand side of the cartoon (pink) shows what happens to cell surface tetherin in the presence of Vpu. Both are internalised and delivered to early endosomes (with tetherin once again exiting lipid rafts along the way) where they associate with one another. This association leads to a shift in the balance between recycling and lysosomal degradation for tetherin, with the majority of tetherin now destined for delivery to MVBs/late endosomes and then to lysosomes (as indicated by the thick black arrows). NB this cartoon illustrates only the effect of Vpu on the fate of internalised tetherin.

Thus i) we find no evidence for a stable interaction between Vpu and endogenous tetherin at the cell surface, ii) the vast majority of endogenous tetherin that is at the cell surface in control cells is in lipid rafts, iii) internalised tetherin is present in non-raft fractions, iv) expression of Vpu in cells expressing endogenous tetherin leads to the loss of tetherin from lipid rafts, v) internalised tetherin enters early endosomes, and late endosomes, in both control cells and cells expressing Vpu, vi) lysosomes are the primary site for degradation of endogenous tetherin in cells expressing Vpu. It is therefore possible that an interaction between Vpu and tetherin at the cell surface initially removes tetherin from lipid rafts and that this is rapidly followed by internalisation of the Vpu:tetherin complex (this rapid removal of the complex would explain why we detect no cell surface biotinylated tetherin co-immunoprecipitating with Vpu). However, published data argue that expression of Vpu does not affect the efficiency with which tetherin is internalised from the cell surface [36,40]. An alternative, and possibly more satisfactory, explanation is that, in both control cells and in cells expressing Vpu, as tetherin is internalised it partitions from ‘raft’ to ‘non-raft’ regions of membrane. Soon after internalisation in cells expressing Vpu, the tetherin encounters Vpu (this is consistent with our observation that incubating biotinylated cells at 37°C for 10 min, to allow internalisation of tetherin, leads to a detectable interaction between Vpu and tetherin) and this interaction modifies the intracellular trafficking route of tetherin such that it is preferentially delivered to the lysosome for degradation rather than being recycled to the cell surface. This is consistent with the reported role of components of the ESCRT (endosomal sorting complex required for transport) machinery in Vpu-dependent degradation of tetherin [20,48]. The Vpu-mediated diversion from the recycling pathway would appear to occur early in the endocytic pathway (as internalised tetherin is detected in both early and late endosomes, whereas Vpu is detected in early, but not late endosomes). The fact that endogenous tetherin is susceptible to lysosomal degradation in control cells (see Figure 6) indicates that it is inefficiently recycled to the cell surface following internalisation in these cells and is consistent with the previously published relatively short half-life of tetherin [40]. Thus the encounter between tetherin and Vpu early in the endocytic pathway simply seems to tip the balance from a situation where some tetherin is delivered to lysosomes for degradation but the majority is recycled to the cell surface into one in which the lysosomal pathway predominates. Thus, Vpu is not removing tetherin from lipid rafts at the cell surface, rather, as tetherin is internalised it exits lipid rafts (NB this is consistent with the concept of lipid rafts as membrane microdomains that are dynamic assemblies of lipids and proteins with constant partitioning of components into and out of the rafts [12,13]) and encounters Vpu; this encounter favours the lysosomal pathway rather than the recycling pathway (see Figure 7). This explanation is also consistent with the observations that it is not necessary for Vpu to be present in lipid rafts for it to down-regulate the level of tetherin expression [49]. Hence Vpu appears to be playing roles in sequestration and degradation of tetherin in both the endocytic and biosynthetic pathways [42].

Endogenous tetherin in HeLa cells is generally detected as a diffuse smear of ~34kDa and above in immuoblot analysis of cell lysates following treatment under reducing conditions and separation by SDS-PAGE (e.g. see Figure 3). This smear corresponds to heterogenous glycosylation of monomeric tetherin. However, we note that the tetherin that co-immunoprecipitates with Vpu is detected as a fairly sharp band corresponding to an ~75kDa molecule following treatment under reducing conditions and separation by SDS-PAGE (e.g. see Figure 1C). We suggest that this molecule represents a particular glycoform (hence the relative sharpness of the band) of an SDS-resistant dimer (hence the increased molecular mass) of tetherin.

Others have recently published that expression of Vpu has no effect on the distribution of tetherin between raft and non-raft membrane microdomains [50]. However, we note that these authors used 0.5% Triton X-100 in their extraction protocol (in contrast to the 1% Triton X-100 used here) prior to sucrose density centrifugation. The experiments giving rise to the data presented in Figure 3A were therefore repeated (at 24 and 48 hours post-transfection to express Vpu-GFP) using 0.5% Triton X-100 instead of 1% Triton X-100. Under these conditions we also saw no effect of Vpu expression on the raft vs. non-raft localisation of tetherin. This serves to highlight the fact that aspects of the subtleties of lipid raft organisation can be dissected by minor differences in detergent concentration in differential extraction procedures.

## Conclusions

We find i) no evidence for an interaction between Vpu and endogenous tetherin at the cell surface, ii) the majority of endogenous tetherin that is at the cell surface in control cells is in lipid rafts, iii) internalised tetherin is present in non-raft fractions, iv) expression of Vpu in cells expressing endogenous tetherin leads to loss of tetherin from lipid rafts, v) internalised tetherin enters early and late endosomes, in both control cells and cells expressing Vpu, vi) lysosomes are the primary site for degradation of endogenous tetherin in cells expressing Vpu. Our studies underlie the importance of studying endogenous tetherin and highlight the fact that recombinant tetherin can be differentially localised between raft and non-raft fractions in different cell types. We propose a model in which Vpu intercepts newly internalised tetherin and diverts it for lysosomal destruction rather than recycling to the cell surface.

## Materials and Methods

### Plasmids

The constructs encoding codon-optimized Vpu and codon-optimized Vpu fused to enhanced GFP (eGFP) (VpuGFP) have been reported previously [34]. C-terminally GFP tagged M2 (A/PR/8/34 strain) was produced by sub-cloning the M2 open reading frame (amplified by RT-PCR from infected cells) into the KpnI/AgeI sites of pEGFP-N1 (Clontech).

### Antibodies

Rabbit polyclonal antiserum directed against the extracellular portion of tetherin has been described previously [51] and was obtained through the NIH AIDS Research and Reference Reagent Program (catalog number 11721; https://www.aidsreagent.org). This was used for immunoblots. A mouse monoclonal antibody (HM1.24) to tetherin [1] was provided by the Chugai Pharmaceutical Company. This was used for immunofluorescence. Additional antibodies against EEA1 (SantaCruz), TGN-46 (gift from Dr. S. Ponnambalam), and LAMP-1 (gift from Dr. A. Toye) were also used for Immunofluorescence.

### Membrane preparations

Detergent resistant membranes were prepared and separated on sucrose density gradients as previously described [47]. 1ml fractions were taken from the sucrose gradients, trichloroacetic acid precipitated, resuspended in sample buffer and separated on a 15% SDS polyacrylamide gel.

### Biotinylation

Surface proteins were labelled on ice with 1mg/ml NHS-SS-biotin in borate buffer (10mM orthoboric acid, 154mM NaCl, 7.2mM KCl, 7.2mM CaCl_2_) for 30 min. Excess biotin was quenched with Glycine, washed with PBS and returned to ice. Cells were either immediately lysed or put with pre-warmed media at 37°C for 10 min and lysed TNE (1% Triton X-100, 150 mM NaCl, 10 mM EDTA) on ice. Lysates where then either incubated immediately with streptavidin beads overnight or separated on a sucrose density gradient. 1ml fractions were pooled as indicated before incubation with streptavidin beads. Proteins were eluted in 100µl of sample buffer.

### Immunoprecipitation

Transfected cells where lysed and interacting proteins were immunoprecipitated using the GFP-TRAP system according to the manufacturer’s (Chromotek) protocol. For the double immunoprecipitation procedure, proteins were eluted in 50µl of elution buffer with 5% Sodium dodecylsulphate for 5 min at 95°C. After centrifugation the supernatant was removed and 1 ml elution buffer was added to dilute the SDS prior to incubation with the streptavidin beads (as above).

### Immunofluorescence

HeLa cells were cultured for immunofluorescence on glass coverslips in DMEM (1% Penicillin-Streptomycin, 10% Foetal bovine serum). After 24 hours, cells were transfected with 3µg GFP-Vpu plasmid using Fugene HD (Promega). At 48hrs, cells were treated with a Lysosomal inhibitor cocktail consisting of 40µM Leupeptin, 40µM Pepstatin, and 4µg/ml of e64D (all from Sigma Aldrich). Immediately prior to fixation, LysoTracker-594 reagent (Molecular Probes) was added to the cell media at a concentration of 500µM, and incubated for 90min in order to internalise the reagent. Cells were fixed at 72hrs with 3% PFA in PBS, and permeabilised using 0.1% Triton X-100. Dual immunolabelling of tetherin was performed by incubating with the HM1.24 primary mouse monoclonal antibody and TGN-46 rabbit polyclonal antibody (see above) for 1 h, washing with PBS, and incubating with Alexa Fluor 633- or 594-conjugated secondary antibody for 0.5 h. Tetherin uptake experiments were performed by incubating cells in PBS on ice for 20min, followed by incubation on ice with HM1.24 antibody in DMEM for 15min. Cells were then placed in DMEM at 37°C for 5min or 10min to allow antibody to be taken up, followed by washing with PBS, fixation with 3% PFA in PBS and antibody labelling with Lamp1 rabbit polyclonal or EEA1 goat polyclonal antibody (see above). Fixed cells were imaged using a confocal laser-scanning microscope (TCS-NT; Leica) equipped with a Kr/Ar laser (488-, 594-, and 647-nm lines) attached to an upright epifluorescence microscope (DMRBE; Leica). All images were collected using a 63× NA 1.4 oil immersion objective and processed with Leica software.

### Inhibitor Assay

HeLa cells were either transfected with plasmid encoding GFP-Vpu as above, or treated with a mock transfection. After 24 hours, cells were treated with Lysosomal Inhibitor Cocktail (as above), Proteasomal Inhibitor (1µM MG132, Sigma Aldrich), or left untreated. At 48hrs, cells were lysed in a buffer consisting of 10 mM Tris-HCl, pH 7.4, and 10 mM EDTA with protease inhibitors (Roche). Proteins were separated on 12 or 15% gels, transferred to PVDF and incubated with primary antibody. Bound primary antibody was detected with HRP-conjugated secondary antibody and chemiluminescence (Roche).
